# Synthesis of gamma irradiated acrylic acid-grafted-sawdust (SD-g-AAc) for trivalent chromium adsorption from aqueous solution

**DOI:** 10.1016/j.hazadv.2024.100427

**Published:** 2024-05

**Authors:** Sobur Ahmed, Abrar Shahriar, Nazia Rahman, Md. Zahangir Alam, Mohammad Nurnabi

**Affiliations:** a Institute of Leather Engineering and Technology, University of Dhaka, 44-50, Hazaribagh, Dhaka, 1209, Bangladesh; b Institute of Nuclear Science and Technology, Bangladesh Atomic Energy Commission, Dhaka, 3787, Bangladesh; c Department of Applied Chemistry and Chemical Engineering, University of Dhaka, Dhaka, 1000, Bangladesh

**Keywords:** Adsorbent, Gamma irradiation, Heavy metals, Isotherm, Kinetics, Sawdust

## Abstract

Water pollution caused by chromium released from tannery is a serious concern to the environment and public health. Chromium removal from tannery effluent is a crying need before discharging to the surface water. In this study, acrylic acid-grafted sawdust was prepared by Tectona grandis sawdust grafting with acrylic acid employing gamma irradiation in the presence of air and Mohr's salt. It was treated with NaOH and the characterization of surface morphology and functional groups of modified sawdust was studied by SEM and FTIR.. The effects of solution pH, adsorbent dosage, adsorption time, and initial Cr(III) ion concentration were investigated by batch sorption studies. The process was found to be pH, temperature and concentration dependent. Langmuir and Freundlich isotherms were applied to realize the adsorption process in depth, and it was found that the Langmuir isotherm model fitted well with experimental data (R^2^ value of 0.983). The maximum monolayer adsorption capacity of acrylic acid-grafted sawdust for Cr(III) from aquous solution was found to be 21.55 mg g^-1^ at 25 °C. Pseudo-first-order and pseudo-second-order kinetic models were employed to analyze the kinetics of the process, and it was found that the experimental process followed the pseudo-second-order kinetic model, i.e. chemisorption. This study revealed that acrylic acid-grafted sawdust has a decent potential for the removal of Cr(III) from tannery effluents.

## Highlights

• Acrylic acid-grafted sawdust (SD-g-AAc) synthesized by γ-irradiation.
• NaOH treated sawdust (t-SD-g-AAc) showed higher adsorption capacity.
• The monolayer adsorption capacity of SD-g-AAc was found 21.55 mg g-1 for Cr(III) at 25 °C.
• Pseudo-second-order model i.e. chemisorption was followed with t-SD-g-AAc for Cr(III) adsorption.

## Graphical abstract

**Figure:**
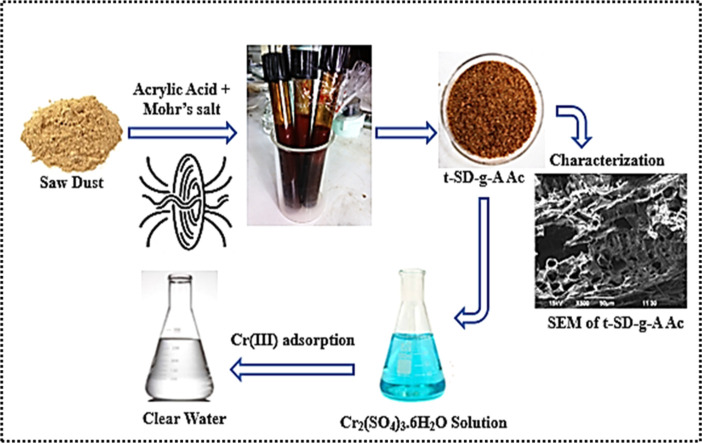

## 1 Introduction

Safe disposal of hazardous industrial wastes is increasingly a global concern (Yang et al., 2018). Tannery in Bangladesh produces roughly 2–3 % of the world's total leather consumption, which is a major source of trivalent chromium in wastewater. The byproducts from meat industry are used in the tannery to provide necessities like leather shoes,apparel, furniture, automoviles, apparel and other leather products (Mahmood Ali, 2023; Fatema-Tuj-Zohra et al., 2023). Every year around 17 million tones of hides and skins are processed by the tanneries worldwide, which released 600 million m^3^ of tannery wastewater (TWW) and 350 million m^3^ of treated wastewater (Rajamoany, 2017). Tannery effluents exhibit high amounts of suspended solids (SS), organic nitrogen, sulfide, and chromium, as well as high levels of chemical oxygen demand (COD) and biological oxygen demand (BOD) (Sundar et al., 2011; Chowdhury et al., 2015). The natural environment is harmed by the elevated SS, DS, BOD, and COD of wastewater due to its high chrome content (Shaibur, 2023; Fatema-Tuj-Zohra et al., 2024). Because it increases the pollution load of already polluted water, it is particularly harmful to aquatic life when this dirty water enters a river or the ocean (Englande et al., 2015). Huge amounts of chromium-containing untreated tannery effluents are released into water bodies, which significantly pollute the environment (Aktar et al., 2023) and raise threats to human health (Nur-E-Alam et al., 2018). Moreover, other heavy meatals, like Mn, Pb, Cu and Fe, may accumulate in the water bodies as a results of several reasons, such as urbanization, population growth, excessive dewatering of aquifers for residential use, and agricultural purposes (Rushdi et al., 2023; Salehi et al., 2021). According to studies, the levels of Cr(III) in the chrome tanning effluent and composite effluents contain 2000–5000 mg l^-1^ and 100–250 mg l^-1^, respectively (Mariliz et al., 2015; Hashem et al., 2020). Plants, animals, and human health are all negatively impacted by chromium levels of tannery effluent (Nur-E-Alam et al., 2020; Hossan et al., 2020). Although Cr(III) is not as hazardous as Cr(VI) at low dosages, it can be catastrophically dangerous in greater numbers (Mitra et al., 2017; Oaishi et al., 2023). High amounts of metals, above the permissible limit of WHO/DoE, were observed in green vegetables cultivated in tannery effluent contaminated soil, particularly for Cr (125.50–168.99 mg/kg Dw) and Cd (0.19–0.83 mg/kg Dw) (Ahmed et al., 2022). Additionally, chromium enters the human body through contaminated chicken feed and is passed along with the food chain (Ahmed et al., 2017). Like many other countries, the discharge level of chromium is 2 mg/L in spent wastewater in Bangladesh (ECR, 2023). Thus, it is vital to reduce chromium from varied industrial wastes, particularly tannery effluent, to preserve the environment.

Different techniques have been developed to solve the issue. Precipitation, membrane filtration, neutralization, ion exchange, floatation, adsorption, etc., are some methods for heavy metals deduction from the effluents, however, adsorption is considered the most economical and active separation technique (Wijaya et al., 2020; Kumari et al., 2021; Ubando et al., 2021; Siggins et al., 2021). Wood and its derivatives are great environmentally friendly materials for removing heavy metals from wastewater because of their plentiful sources, ability to defense the environment, and natural adsorption capabilities (Mohanapriya et al., 2023; Shahjahan et al., 2022). Biosorption is a process by which biological material removes chemicals from a solution (Ramesh, 2023). According to Kanamarlapudi et al. (2018), the mechanism of biosorption consists of the physical binding of the metal ions (electrostatic force of attraction or Van der Waals force of attraction) or the chemical binding (exchange of ions) of the metal ions, chelation, complexation, reduction, and precipitation. It is the physical or chemical binding that allows chemical species to be absorbed onto natural material from an aqueous solution (Syeda et al., 2022). Previous studies revealed that various bio-adsorbents such as mango peel, potato peel, citrus peels, coconut copra meal, sawdust, corn cob, rice husk, wheat bran, grape bagasse, tree fern, orange wastes, and fly ash and blast furnace slag had interesting heavy metal adsorption properties (Nguyen et al., 2018; Malik et al., 2016; Pakade et al., 2019; Zadeh et al., 2020; Jaouadi, 2020). The majority of these methods have drawbacks, such as high initial and ongoing costs. Therefore, it is necessary to create accessible, low-cost materials with the ability to inexpensively absorb Cr and other contaminants. Adsorption is the process by which molecules, atoms, and ions from a gas, liquid, or dissolved solid stick to the surface of adsorbents to produce an adsorbate coating (Tatarchuk et al., 2023). Research has been done on the removal of pollutants including chromium Cr (III) from tannery effluent by batch adsorption method, which uses a variety of low-cost adsorbents (Khan, 2023). The main objective of the study was to find a feasible, easily accessible, reasonably priced, ecologically benign, and extremely effective adsorbent.

Modification of sawdust was carried out and shown to have higher adsorption capabilities (De Gisi, 2016; Gunatilake, 2016; Wang et al., 2020; Yu et al., 2020; Zakaria et al., 2009). Modification through grafting of appropriate monomers onto the sawdust can be performed in different techniques, including plasma treatment (Handrof et al., 2020), ionizing radiation (Chu et al., 2021), and ultraviolet light irradiation (Timmermann et al., 2015). However, radiation-induced grafting allows for the broad penetration of radiation into the polymer matrix and the uniform fast synthesis of radicals to commence grafting without the use of any harmful chemical initiator (Jhimli et al., 2017; Samani and Toghraie, 2019). As a new technology emerge, there is no research performed on the application of gamma irradiation on grafting of acrylic acid in sawdust, their benefits and potential drawbacks. Considering the availability of sawdust, a waste generated from the woodwork, *Tectona grandis* (Tick) sawdust could be a very inexpensive adsorbent to utilize. The synthesis of acrylic acid grafted sawdust via γ-irradiation, their modification, and employment as a potent adsorbent for the removal of Cr(III) ions from aqueous solution are the main aims of this work.

## 2 Materials and method

### 2.1 Materials

Sawdust of *Tectona grandis* was collected from a sawmill at Rayerbazar, Dhaka, Bangladesh, Acrylic acid (AAc), methanol, acetone, and nitric acid (65 %) were purchased from Merck, India. Hydrogen peroxide (33 %) and Mohr's salt were obtained from BDH, UK. Chromium sulfate (Cr_2_(SO_4_)_3_·6H_2_O) was purchased from Qualikems Fine Chem., India to prepare a standard chromium(III) solution.

### 2.2 Instruments

90 kCi Cobalt-60 Batch Type Panoramic Irradiator (BRIT, India) was applied as the source of γ-irradiation. Fourier-transformed infrared spectroscope (FTIR) (8400S Shimadzu, Japan) and a scanning electron microscope (SEM) (JSM-6490LA, JEOL, USA) were used to characterize the prepared adsorbent. Atomic absorption spectroscopy (AAS) (AA-6800 Shimadzu, Japan) was used to estimate the concentration of chromium.

### 2.3 Synthesis of acrylic acid-grafted sawdust (SD-g-AAc) by gamma irradiation

**Fig. 1:**
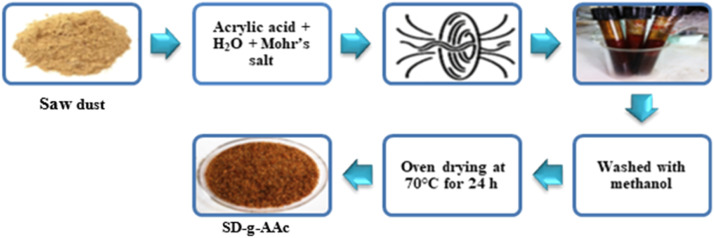
Flowchart of SD-g-AAc preparation by gamma irradiation.

**Fig. 2:**
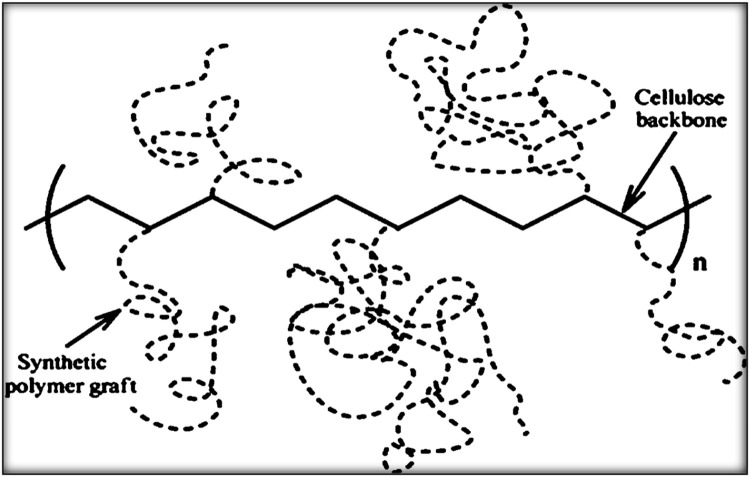
A schematic representation of cellulose graft copolymer.

Collected sawdust was cleaned with tap water followed by de-ionized (DI) water. Then after drying 2 d in the sun, the sawdust was dried in an oven at 65 °C for 24 h. The untreated sawdust (u-SD) thus obtained was preserved in a sealed container for further use. In this investigation, 60 mesh sawdust samples were employed. The ungrafted sawdust samples, u-SD weighing W_0_ (2 g) were taken in each of the separate four test tubes along with 20 mL of prepared acrylic acid solution of different concentrations. The 20 mL solution was made by adding 2, 4, 6, and 8 mL acrylic acid in 18, 16, 14, and 12 mL of distilled water, respectively along with 0.6 g Mohr's salt inhibitor for every experiment. Then the test tubes’ mouths were tightened with their cap, homogenized well, and prepared for irradiation and grafting. After that, the tubes were exposed to gamma radiation for 4 h at ambient temperature using a Co-60 source at various radiation dosages (10 KGy to 50 KGy). After being exposed to radiation, the sawdust samples were cleaned with methanol to eliminate homopolymers, dried for 24 h at 70 °C in an oven, and then chilled in desiccators (Fig. 1). The resulting material was sealed in an airtight container and labeled as SD-g-AAc. Among the methods of modification of polymers, graft copolymerization offers an attractive and versatile means of imparting a variety of functional groups to a polymer (Bhattacharya and Misra, 2004). A schematic representation of cellulose graft copolymer is shown in Fig. 2.

The grafting yield was calculated by Eq. (1):

(1) $$Degreeofgrafting\left(\%\right)=\frac{W_{1}-W_{0}}{W_{0}}\times 100$$

Where *W_0_* and *W_1_* stand for the mass (g) of u-SD and SD-g-AAc, respectively.

### 2.4 Treatment of SD-g-AAc with aqueous NAOH

SD-g-AAc (1.0 g) were mixed with NaOH solution (0.01 M, 30 mL) and agitated for 30 min with continuous stirring. The treated sawdust was then repeatedly cleaned in DI water and dried in an air oven at 70 °C for 24 h to obtain treated acrylic acid-grafted sawdust (t-SD-g-AAc).

### 2.5 Synthesis of adsorbent

**Fig. 3:**
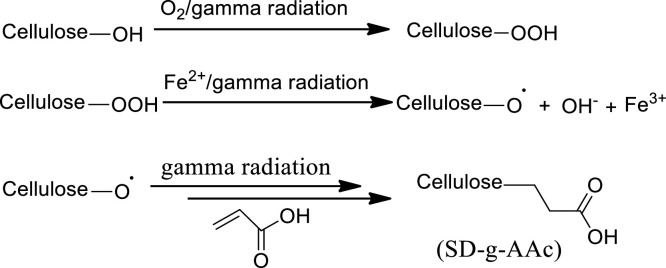
Mechanism of the formation of SD-g-AAc.

Sawdust mostly consists of cellulose, lignin, tannins, and other phenolic chemicals and the lignin molecule is composed of syringaldehyde and vanillin (Mota et al., 2015). The sawdust sample was grafted with acrylic acid by mutual irradiation method, where the substrate and monomer mixture was directly irradiated with gamma radiation in the presence of air. The mechanism is shown in Fig. 3 (Roy et al., 2009) and it was assumed that in the presence of air cellulose hydroperoxide (Cellulose-OOH) was formed in the first step, which should be dissociated to form cellulose free radical (Cellulose-O-) and hydroxyl free radical (OH**^-^**). Hydroxyl free radical would next react with AAc monomer to form a homopolymer.

#### 2.5.1 Optimization of radiation dosage on grafting yield

Fig. 3 illustrates the impact of radiation dosage on the degree of AAc grafting onto sawdust, revealing that the percentage of grafting increases as radiation dosage increases. At 10 KGy radiation dosages, the percentage of grafting was only 8.15 % whereas; it was 27.44 % at 50 KGy. However, it was very difficult to wash the material obtained from 50 KGy due to extreme stickiness, and, therefore, 40 KGy radiation was chosen as the optimum radiation dosage.

#### 2.5.2 Optimization of monomer concentration

**Fig. 4:**
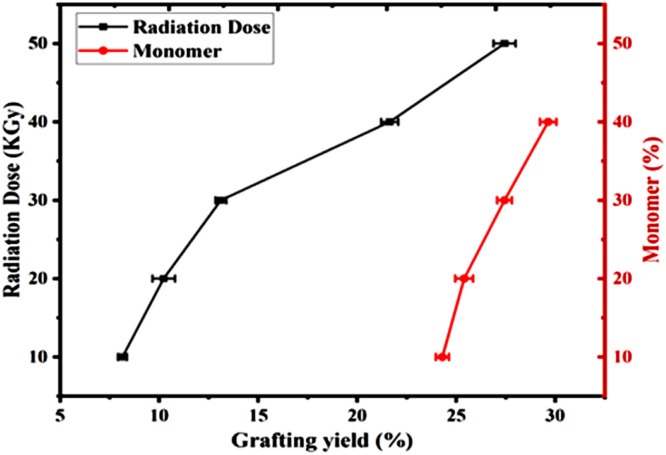
Effects of monomer concentration and radiation dosage on grafting yield.

Four distinct grafting experiments were conducted using 10, 20, 30, and 40 % AAc concentration at 40 KGy radiation dosage at room temperature for 4 h and the outcomes are displayed in Fig. 4. It is found that the grafting yield increased with the increasing the concentration of monomer. However, an excess amount of monomer (more than 30 %) formed a lumpy acrylic acid homopolymer during the process which was too difficult to remove. Since the percentages of grafting were almost the same for 10 % and 20 % concentrations, the 10 % monomer concentration was considered optimum for the irradiation process.

### 2.6 Adsorption studies

To find the Cr(III) absorption capacity of the SD-g-AAc or t-SD-g-AAc, each adsorbent (2.0 g) was mixed with a metal ion solution (50 mL) in a conical flask and agitated for 90 min at 80 rpm in an orbital shaker 25 °C. Whatman 41 filter paper was used to filter the mixture and metal content was determined by examining the filtrate with AAS. The percentage of removal and adsorption capacity (*q_e_*) were determined following the Eqs. (2) and (3), respectively.

The effect of pH was studied using Cr(III) solution (50 mL) in several conical flasks adjusting pH at 2.5, 3.0, 3.5, 4.0, and 4.5 with dilute hydrochloric acid. Each flask was filled with adsorbent (2.0 g) and agitated for 90 min 80 rpm at room temperature. Following filtration with the Whatman 41 filter paper, the chromium concentration was determined using AAS and adsorption capacity was calculated.

The adsorbent (2.0 g) was added with Cr(III) solution (50 mL) in each conical flask at pH 4.5 controlling concentrations of 200, 300, 400, 600, and 800 ppm, and the mixture was shaken to observe the impact of concentration and contact time. 2 mL aliquots were drawn at various intervals to measure the metal concentration with AAS.

(2) $$\%ofremoval=\frac{C_{0}-C_{e}}{C_{0}}\times 100$$

Where ‘C_0_ and C_e_’ denote the concentration (mg l^-1^) of Cr(III) at initial and equilibrium, respectively.

(3) $$Adsorptioncapacity,q_{e}=\frac{(C_{0}-C_{e})\times V}{W}$$

where ‘q_e_’ denotes the adsorption capacity at equilibrium (mg g^-1^ of adsorbent), ‘C_0_ and C_e_’ represent the concentrations (mg l^-1^) of Cr(III) at initial and equilibrium, respectively, whereas ‘V’ and ‘W’ indicate volume of solution (L), and weight of the adsorbent (g), respectively.

## 3 Results and discussion

### 3.1 Characterization of adsorbent

#### 3.1.1 FTIR analysis

**Fig. 5:**
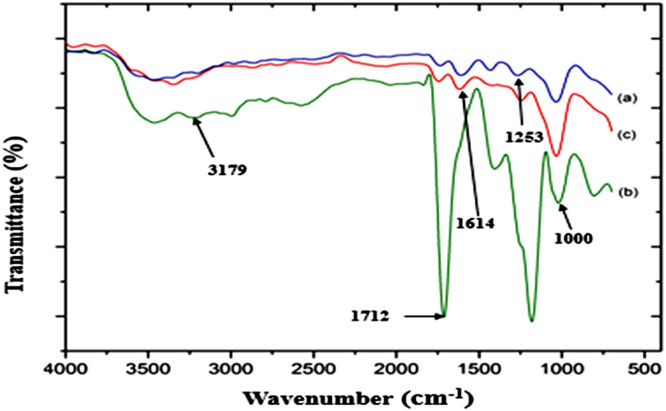
FTIR spectra of (a) u-SD, (b) SD-g-AAc, and (c) t-SD-g-AAc.

In this study, Mohr's salt was utilized as a reducing agent to decrease the homopolymer production, which delays the synthesis of acrylic homopolymer by giving up an electron to the hydroxyl free radical and converting it to hydroxyl ion (OH-) (Roy et al., 2009; Bhattacharya and Misra, 2004). The occurrence of chemical changes during the grafting process of sawdust was evident in the FTIR spectroscopic analysis (Fig. 5).

FTIR spectra of u-SD, SD-g-AAc, and t-SD-g-AAc demonstrated similar vibrational frequencies at 3300–3500(br), 3179, 1712, 1614, 1253 and 1000 cm^-1^, which are attributed to the -OH stretching, C—H stretching, C=O stretching, C=C stretching, C—O-H bending and C—O stretching, respectively. However, SD-g-AAc showed intense vibrational frequencies compared to those for raw sawdust. The strong C=O vibrational frequencies at 1712 cm^-1^ indicated C=O bond of –COOH group of AAc and was successfully grafted onto the main chain of sawdust. All the differences happened between the untreated raw and treated sawdust confirmed the acrylic acid grafting onto the sawdust. Again, after treating of t-SD-g-AAc with sodium hydroxide solution the strong carbonyl vibration almost disappeared due to chemical reaction on the surface.

#### 3.1.2 SEM analysis

**Fig. 6:**
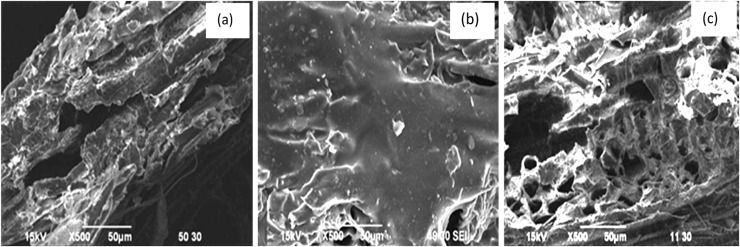
SEM micrograph of (a) u-SD, (b) SD-g-AAc and, (c) t-SD-g-AAc.

Surface morphologies of u-SD, SD-g-AAc, and t-SD-g-AAc were investigated using SEM and it was evident that u-SD consists of cavities of different shapes and sizes with high surface roughness (Fig. 6. (a)), while SD-g-AAc surface (Fig. 6. (b)) was almost covered with acrylic homopolymers. After NaOH treatment, the homopolymers were removed and due to the scouring effect, t-SD-g-AAc had a clean surface with cavities and roughness (Fig. 6. (c)).

#### 3.1.3 X-ray diffraction analysis

**Fig. 7:**
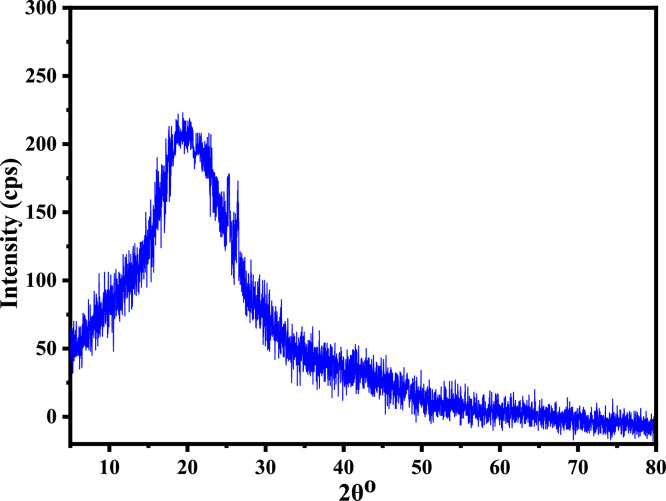
XRD pattern of t-SD-g-AAc.

Saw dust is a polycrystalline structure materials, which contain crystalline cellulose and amorphous lignin region (Kim et al. 2012). The XRD pattern of t-SD-g-AAc is shown in the Fig. 7. It was observed a wide hump in the range of 2θ =10–30° and a peak at 2θ = 20.86°, which signify the amorphous structure with a high degree of disorder and damage of natural structure of cellulose cell wall due to grafting with acrylic acid monomer and later on treatment with NaOH (Zhang et al., 2019).

### 3.2 Adsorption of Cr(III) by modified sawdust

#### 3.2.1 Removal of Cr(III) by u-SD, SD-g-AAc and t-SD-g-AAc

To compare the percentage of chromium(III) removal from solution by u-SD, SD-g-AAc, and t-SD-g-AAc, Cr(III) solutions (50 mL) of three distinct concentrations (200, 300, 400, and 600 ppm) were mixed with the adsorbent (2.0 g) separately, and agitated at 25 ºC for 24 h. After the mixtures were filtered with whatman filter paper, the Atomic absorption spectroscopy (AAS) was used to measure the concentration of chromium.

**Fig. 8:**
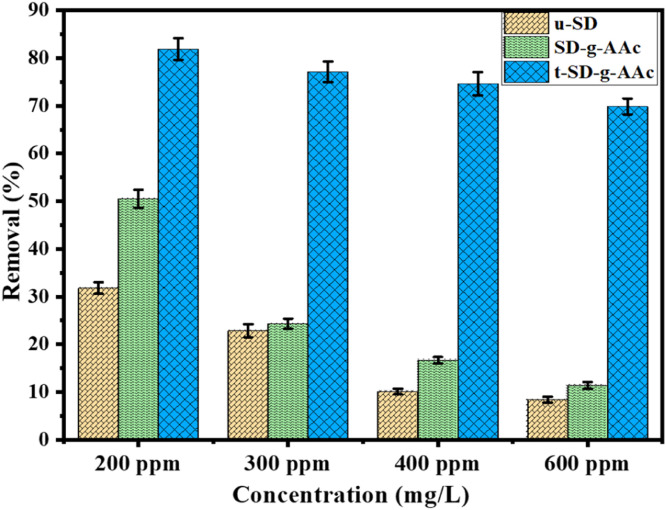
Percentages of Cr(III) removal by u-SD, SD-g-AAc and t-SD-g-AAc.

The percentage removal for the adsorbents is presented in Fig. 8 and it was evident that t-SD-AAc showed the highest percentage of adsorption, e.g. in the case of 200 ppm Cr(III) solution the removal was 31.80 %, 51.00 %, and 81.88 % with the adsorbent of u-SD, SD-g-AAc, and t-SD-g-AAc, respectively. Since t-SD-AAc demonstrated promising adsorption capacity, further studies were carried out using this adsorbent.

#### 3.2.2 Effects of pH

**Fig. 9:**
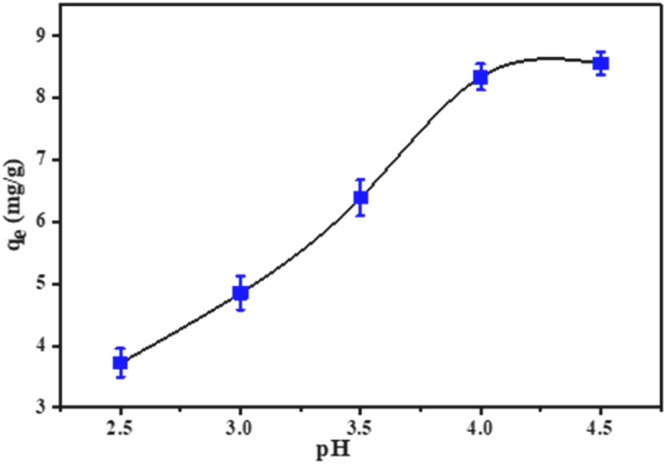
Effects of pH on adsorption.

At room temperature, the impact of pH on Cr(III) adsorption on t-SD-AAc was investigated. Cr(III) solution (50 mL) was taken in various conical flasks and the pH was adjusted to 2.5 – 4.5. Then the adsorbent (2.0 g) was mixed with each solution, and the flasks were agitated at 80 rpm for 24 h. It was observed that the adsorption capacity increased with the increasing pH value, and reached 8.5 mg g^-1^ at pH 4.5 (Fig. 9). The experiments was conducted upto pH 4.5, since the Cr(III) was forming insoluble hydroxide beyond this pH.

The surface characteristics of the adsorbent and the species distribution of chromium in water can explain the effect of pH on adsorption. The most prevalent Cr(III) cation species in acidic pH are Cr^3+^, Cr(OH)^2+^, and Cr(OH)_3_. Decreases in pH also lead to protonation of the adsorbent surface, which reduces the electrostatic interaction between the Cr(III) species and the adsorbent surface and thus lowers the adsorption percentage. However, the adsorbent surface becomes less protonated and draws cationic Cr(III) ions more strongly as pH rises. This led to an overall increase in Cr(III) adsorption at higher pH levels and a decrease at lower pH values (Bedemo et al., 2016). All of the batch investigations in this investigation were carried out at pH 4.5 in order to achieve the maximum adsorption capacity without turbity or Cr(III) precipitation, since Cr(III) can precipitate and separate as Cr(OH)_3_ from aqueous solution at pH>6.0 (Santos et al., 2012; Ahamed et al., 2020).

#### 3.2.3 Effect of concentration and contact time

**Fig. 10:**
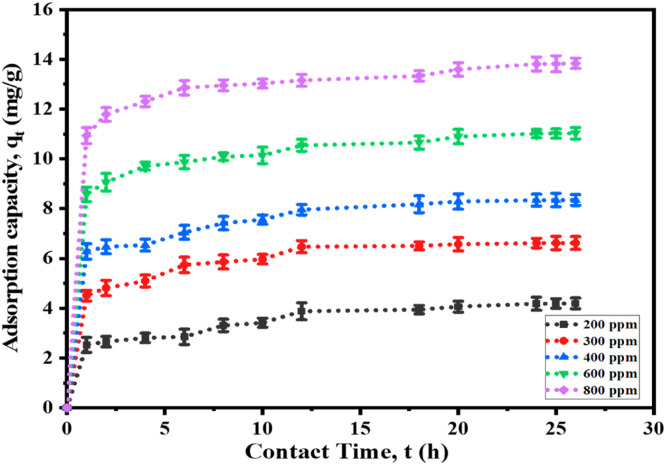
The effect of concentration and contact time on adsorption capacity.

Using Cr(III) solution (50 mL) in a conical flask and adsorbent (2.0 g), the effects of concentration and time on adsorption were inspected. The study utilized pH 4.5 and five concentrations (200–800 ppm) to evaluate the connection between concentration and time. An orbital shaker was used to shake the samples at a speed of 80 rpm at 25 °C. After a specific time, the adsorbent was separated by filtering using the Whatman 41 filter paper, and the filtrates were later exposed to an AAS analysis to assess the adsorption capacity. It was found that within 24 h the adsorption reached almost the highest value (Fig. 10). The initial high adsorption capacity was most likely brought about by the increased surface area of t-SD-AAc being available. The transfer rate from the adsorbent's outside to interior sites influences the rate of absorption as the adsorption sites are exhausted (Rafatullah et al., 2009).

However, it was obvious that the adsorption capacity increased with the rise in metal concentration. The maximal adsorption capacity was observed at 13.81 mg g^-1^ at 800 ppm solution. The resistance of mass transfer between the solution and the adsorbent surface was overcome by the driving force given by concentration in the bulk solution (Yang et al., 2013). Consequently, a larger initial concentration of Cr(III) ions led to an increase in the Cr(III) adsorption.

#### 3.2.4 Adsorption isotherms

Equilibrium adsorption isotherm displays how adsorbates are distributed throughout the adsorbent surface and liquid phases at equilibrium (Argun et al., 2007) and thus design an adsorption system. Utilizing Freundlich and Langmuir isotherm models, the mechanism of t-SD-g-AAc's adsorption for the removal of Cr(III) was quantified and studied. The experimental data was fitted to the Langmuir adsorption isotherm using the following linear expression Eq. (4).

(4) $$\frac{C_{e}}{q_{e}}=\frac{1}{q_{m}b}+\frac{1}{q_{m}}C_{e}$$

Where ‘Ce’ denotes the concentration at equilibrium (mg l^-1^), ‘q_m_’ represents the monolayer saturation adsorption capacity of the adsorbent (mg g^-1^), ‘*q_e_’* is the adsorption capacity at equilibrium, and ‘b’ is the Langmuir adsorption constant (L mg^-1^). Hence, a plot of *C_e_/q_e_* vs *C_e_* should be a straight line with a slope (*1/q_m_*) and an intercept as *1/q_m_b*. The calculated values of constants *q_m_* and *b* were reported in Table 1.

**Table 1:** Parameters of isotherms for adsorption of Cr(III) on t-SD-AAc at 25 °C.

**Langmuir isotherm**				**Freundlich Isotherm**		
***q_m_* (mg g^-1^)**	**b (L mg^-1^)**	**_R2_**	**R_L_**	**K_F_**	n	**R^2^**
21.55	0.0058	0.983	0.117	0.1396	1.116	0.871

The separation factor, *R_L_* (a dimensionless equilibrium parameter) was computed using the Eq. (5) for a more thorough examination of the Langmuir isotherm (Mondal and Chakraborty, 2020).

(5) $$R_{L}=\frac{1}{1+b.C_{m}}$$

**Fig. 11:**
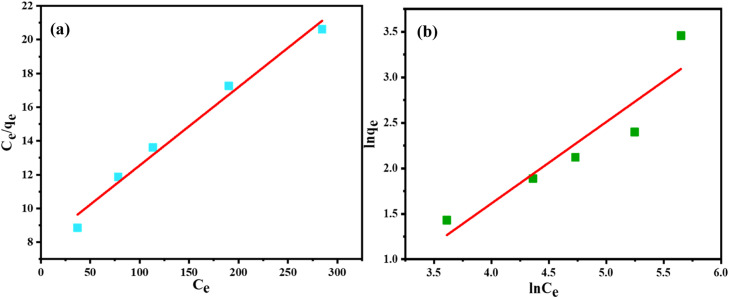
(a) Langmuir, and (b) Freundlich isotherm models.

Where ‘*C_m_’* is the maximum initial concentration (mg l^-1^) of Cr(III) ion. When R_L_ value is between 0 and 1, adsorption will be favourable, whereas it will be unfavorable if R_L_>1, and linear R_L_ is 1. The adsorption process cannot be reversed if R_L_ =0 (Rafatullah et al., 2009). The correlation coefficient (R^2^=0.983) and linear relationship between C_e_/q_e_ and C_e_ showed that the parameters of Langmuir isotherm were in good agreement (Fig. 11. (a)). The value of R_L_ 0.117 (0<R_L_<1) indicated the favourable adsorption process.

The Freundlich isotherm model depicts the multilayer adsorption process that leads to heavy metal adsorption on a heterogeneous surface. The Eq. (6) represents the expression of this model.

(6) $$Inq_{e}=Ink_{F}+\frac{1}{n}InC_{e}$$

where, where "n" is an empirical parameter and $\text{‘}{K_{F}}^{\prime }$ is the sorption capacity (mg g^-1^). The plot of ln $q_{e}$ vs ln $C_{e}$ shown in Fig. 10(b) was made using the experimental data.

The Freundlich isotherm's correlation coefficient (R^2^=0.871) shows how well the Langmuir equation captured the adsorption process. The uniform distribution of active sites on the t-SD-g-AAc surface is the cause of this. Using the Langmuir plot, the maximum capacity (q_max_) of t-SD-AAc at room temperature (25 °C) was revealed to be 21.55 mg g^-1^.

#### 3.2.5 Adsorption kinetics

The rate at which Cr(III) ions move from the aqueous to the solid phase and the amount of time needed to bring the phases into equilibrium have been determined using the adsorption kinetics (Božić, et al., 2013). Using pseudo-first-order (PFO) and pseudo-second-order (PSO) kinetic models, the kinetics of Cr(III) ion adsorption on t-SD-AAc were examined. Using Eq. (7), the PFO model was investigated. (Dizge and Tansel, 2011).

(7) $$\log\left(q_{e}-q_{t}\right)=\log q_{e}-\left(\frac{k_{1}}{2.303}\right)t$$

Where ‘*q_t_’* represents the quantity of solute adsorbed at any given time (mg g^-1^), ‘*k_1_’* is the adsorption constant and ‘*q_e_*’ is the solute adsorbed at equilibrium (mg g^-1^).

**Fig. 12:**
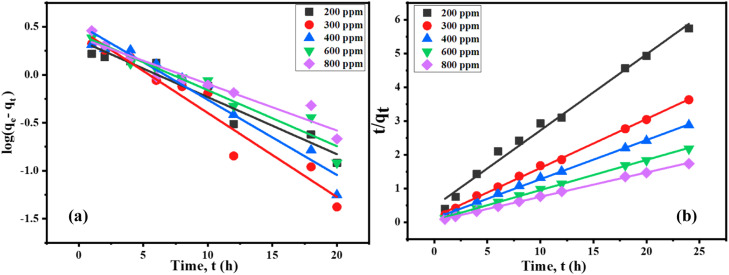
(a) PFO and (b) PSO kinetic plots for Cr(III) adsorption on t-SD-g-AAc at 25 °C.

The PFO kinetic model was found to have poor match to the experimental data (Fig. 12.(a)). The data obtained by applying Eq. (8) to the PSO adsorption kinetics model (Kan et al., 2017) was then tested.

(8) $$\frac{t}{q_{t}}=\left(\frac{1}{k_{2}q_{e}^{2}}\right)+\left(\frac{1}{q_{e}}\right)t$$

where, ‘*q_t_, and q_e_’* denote adsorption capacities at time *t* and equilibrium, and ‘*k_2_*’ is constant. The plot's slope and intercept can be used to calculate the values of qe and k_2_. The two kinetic models' various parameters are shown in Table 2. Plotting t/q_t_ vs t (Fig. 12. (b)) revealed a linear connection with a remarkable correlation coefficient (R^2^) value >0.989, exceeding the PFO kinetic model. Moreover, there is a strong correspondence between the observed values of q_e_ at different dosages and the theoretical values of q_e_ for the PSO kinetic model. According to this model, chemisorption-based surface adsorption, where removal from a solution is caused by physicochemical interactions between the two phases, is the rate-limiting step (Wang et al. 2007). Consequently, the experimental findings confirmed the adsorption process was accurately characterized by the PSO kinetic model followed chemisorption.

**Table 2:** Adsorption kinetics model rate constant of t-SD-g-AAc.

***C_0_* (mg L^-1^)**	***q_e_* (exp.) (mg g^–1^)**	**PFO rate constant**			**PSO rate constant**		
		***q_e_* (theor.) (mg g^-1^)**	***k_1_* (h^–1^)**	***R^2^***	***q_e_* (theor.) (mg g^-1^)**	***k_2_* (g mg^-1^ h^–1^)**	***R^2^***
200	4.18	2.29	0.137	0.938	4.44	0.108	0.989
300	6.61	2.99	0.201	0.943	6.89	0.136	0.998
400	8.34	3.33	0.180	0.951	8.63	0.116	0.998
600	11.02	2.64	0.134	0.936	11.17	0.138	0.999
800	13.81	2.45	0.111	0.925	13.91	0.147	0.999

### 3.3 Possible mechanism of adsorption

**Fig. 13:**
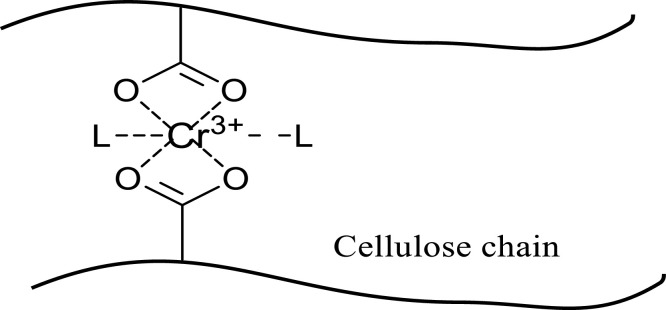
Possible interaction of Cr(III) onto t-SD-g-AAc.

Chromium ions typically combine to create hexacoordinate complexes. The synthesized t-SD-g-AAc used in this investigation has carboxylate groups from the grafted acrylic acids, which are likely responsible for the formation of hexacoordinate complexes by removing Cr(III) from the solution (Fig. 13).

### 3.4 Comparison with reported adsorbents

Scholars have directed their attention towards various adsorbents that show promise in the elimination of metal ions. Table 3 presents a comparison of Cr(III) adsorption on acrylic acid-grafted sawdust (t-SD-g-AAc) with various adsorbents that have been previously described.

**Table 3:** Comparison of reported adsorbents for Cr(III) in literature with the present study.

**Adsorbents**	**pH**	**Time (h)**	***q_max_* (mg g^-1^)**	**References**
Leonardite	4–5	4.0	75.2	Lao-Luque et al. (2014)
Rice husk	5.0	1.0	30	Sobhanardakani et al. (2013)
PNS*	5.0	4.0	104.82	Fatema-Tuj-Zohra et al. (2022)
PACF⁎⁎	4.5	6.0	81.30	Aktar et al. (2023)
Peat	–	–	18.75	Henryk et al. (2016)
Coconut fiber	–	–	19.21	
Vesicular basalt rock	6.0	8.0	0.976	Alemu et al. (2019)
Grainless corn stock	4.6	3.0	7.3	Bellu´et al. (2008)
Chitosan flakes	3.8	2.0	30.03	Pietrelli et al., 2020
Modified peanut husks	4.0	1.0	7.67	Li et al. (2007)
Sawdust	4.0	6.0	5.52	
Oats straw			9.93	
Agave bagasse	4.0	–	10.84	Bernardo et al. (2009)
Sorghum straw			6.96	
Sawdust grafted Poly-(methacrylic acid)	7.0	4.0	36.63	Anirudhan and Radhakrishnan (2007)
Graphene oxide	4.5	0.30	366.3	Ahmed et al. (2021)
Wallnut shell	3.5	–	8.01	Jain et al. (2009)
Hazelnut shell	3.5	–	8.28	
Almond shell	3.2	–	3.4	
Sawdust	3.0	–	1.47	Alvarez-Ayuso et al. (2003)
Rice husks	3.0	–	0.63	
Coirpith	3.0	–	0.16	
Vermiculite	3.0	–	0.26	
Copyrolyzed rice husk char	4.5	48	9.23	Dias et al. (2020)
Acrylic acid-grafted sawdust	4.5	24	21.55	Present study

⁎ PNS**=** Pyrolized activated Peanut shel.

⁎⁎ PACF= Pyrolized activated coconut fiber.

## 4 Conclusions

The study demonstrated the effectiveness of acrylic acid grafted Tectona grandis sawdust surfaces in removing chromium ions from an aqueous solution. The Teak tree (Tectona grandis) sawdust was modified by grafting acrylic acid with gamma irradiation. The impact of radiation dosage and monomer concentration on the degree of grafting was investigated. FTIR and SEM were used to characterize the surface morphology and functional groups of modified sawdustWhen compared to previously reported other comparable adsorbents, the produced t-SD-g-AAc was shown to be quite effective when used to remove Cr(III) from the aqueous solution. The effects of pH on t-SD-g-AAc adsorption demonstrated that the procedure works best at pH 4.5. The pseudo-second-order model provided the best fit for the kinetics data, and the outcome showed that the t-SD-g-AAc adsorbent is very effective at removing chromium from aqueous solution at different concentrations. The adsorbents of sawdust showed tremendous potential to minimize problem generated by tannery wastwater as well as wood industry. The overall study emphasized the efficiency of t-SD-g-AAc as an inexpensive, effective, eco-friendly and viable adsorbent with high adsorption capacity for the removal of chromium. However, the study has some limitation. One important limitation of the study is removing unreacted monomer from grafted saw dusts. Moreover, the lower numbers were not taken into consideration while dosing. Hence, in the future, the optimized values can be found using a variety of Design of Experiment models, including the Pareto Optimization Model and Response Surface Methodology. Further studies are required on more efficient, cost-effective and progressively recyclable methods for its application in real tannery effluents, reusability and contribution in circular economy. Finally, more research in the future on systematic application of chrome remediation in the environment is essential.

## Funding

No particular grant from a public, private, or nonprofit organization was obtained for this work.

## CRediT authorship contribution statement

**Sobur Ahmed:** Writing – review & editing, Writing – original draft, Visualization, Methodology, Investigation, Conceptualization. **Abrar Shahriar:** Writing – original draft, Software, Data curation. **Nazia Rahman:** Writing – review & editing, Validation, Resources, Conceptualization. **Md. Zahangir Alam:** Writing – review & editing, Validation, Supervision, Funding acquisition. **Mohammad Nurnabi:** Writing – review & editing, Supervision, Project administration.

## Declaration of competing interest

The authors declare that they have no known competing financial interests or personal relationships that could have appeared to influence the work reported in this paper.

## Data Availability

Data will be made available on request.
